# Impact of an AI workshop on knowledge and attitudes toward AI in scientific publishing among surgeons at an international abdominal wall surgery congress

**DOI:** 10.3389/jaws.2026.17401

**Published:** 2026-09-14

**Authors:** Mireia Verdaguer-Tremolosa, Georgia Kotoreni, Simon Hoggart, Manuel López-Cano

**Affiliations:** 1 Department of Surgery, Vall d’Hebron Teaching Unit of Medicine, Universitat Autònoma de Barcelona, Barcelona, Spain; 2 Department of General and Gastrointestinal Surgery, Abdominal Wall Surgery Unit, Division of General and Gastrointestinal Surgery, Vall d’Hebron University Hospital, Barcelona, Spain; 3 Department of General Surgery and Emergency Department, General Clinic of Thessaloniki, Thessaloniki, Greece; 4 Frontiers Media SA, Lausanne, Switzerland

**Keywords:** abdominal wall surgery, artificial intelligence, generative AI, hernia surgery, manuscript writing

## Abstract

**Background:**

Generative artificial intelligence (AI) tools are increasingly used for scientific writing, literature synthesis, and peer-review, yet their responsible use requires awareness of hallucinations, fabricated references, confidentiality risks, authorship limitations, and disclosure requirements.

**Objective:**

To evaluate the perceived impact of a focused educational workshop on knowledge and attitudes toward AI in scientific publishing among participants in an abdominal wall surgery workshop.

**Methods:**

An anonymous pre-post survey study was conducted during the JAWS Workshop 2026 on Artificial Intelligence in abdominal wall surgery. Participants voluntarily completed a 10-item baseline survey before two educational presentations and a 10-item post-workshop survey immediately afterwards. Responses were analysed descriptively. Because the questionnaires were not identical, formal paired analysis was restricted to the common ordinal knowledge item using anonymous participant codes.

**Results:**

Thirty-nine participants completed the baseline survey and 41 completed the post-workshop survey; 34 anonymous codes could be matched. Before the workshop, 48.7% reported basic knowledge, 35.9% intermediate knowledge, 5.1% advanced knowledge, and 10.3% no knowledge. AI tools were used occasionally, frequently, or systematically by 89.7% of respondents. The most frequent uses were language editing (74.4%), manuscript structuring (46.2%), literature summarization (46.2%), and data analysis or interpretation (41%). After the workshop, intermediate or advanced self-reported knowledge was observed in 68.3% of respondents. In matched analysis, self-reported knowledge scores improved in 18/34 participants (52.9%; Wilcoxon signed-rank, p = 0.0005). Post-workshop, 92.7% agreed or strongly agreed that they better understood AI limitations, 97.6% would almost always or always verify AI-generated references, 85.4% supported disclosure of AI use in manuscripts, and 87.8% considered society guidelines recommended or essential.

**Conclusion:**

AI use for scientific activities was common among workshop participants, and a brief targeted educational intervention was associated with improved self-reported knowledge and support for responsible AI use. Scientific societies and journals may play an important role in promoting the responsible integration of AI into scientific publishing through focused educational initiatives.

## Introduction

Artificial Intelligence (AI) has been framed in surgery as both an opportunity and a challenge, as it may augment surgeons’ decision-making while introducing concerns regarding validation, interpretability, bias, safety, and accountability [1]. Within abdominal wall surgery, recent systematic review evidence suggests that machine learning and deep learning have been explored for hernia-related tasks including operative video recognition, image-based prediction and postoperative outcome modeling but the field remains early and heterogeneous [2].

In parallel with these clinical applications, large language models (LLMs) and other generative AI tools have also entered academic practice. Evidence synthesis has identified potential benefits for scientific writing, literature review, data analysis, code generation and education but has also emphasized risks related to ethics, transparency, legal responsibility, bias, plagiarism, hallucination and inaccurate citations [3]. Scientific writing and peer-review are cornerstones of the academic world, and generative AI may support both when used transparently, critically and under full human accountability [4]. However, important concerns remain: privacy, misinformation, lack of transparency, authorship, plagiarism, and overdependence. AI-generated content therefore requires critical review by human experts before being used in clinical or scholarly contexts [5]. Empirical studies have demonstrated that LLMs may generate incorrect or fabricated references when asked to support systematic-review-type tasks with hallucination rates that remain high enough to preclude unsupervised use for evidence synthesis [6, 7].

Editorial and publishing organizations have responded by developing policies on authorship, disclosure and accountability. The World Association of Medical Editors (WAME) recommends that generative AI tools cannot be authors because they cannot take responsibility for the work and that their use should be disclosed when they assist with scholarly manuscripts [8]. In peer-review, generative AI may offer efficiency gains, but it also raises concerns about confidentiality, transparency and preservation of expert human judgment [9, 10].

Residents and fully trained surgeons are already using AI tools to write manuscripts, respond to reviewers, and prepare peer-reviews [11]. However, the rapid adoption of AI has outpaced formal training, contributing to an educational gap in scientific publishing [12]. This creates a need for focused educational interventions that address both practical utility and responsible conduct. The present study evaluated the perceived impact of an AI knowledge workshop focused on scientific publishing among abdominal wall surgeons within an active scientific society with its own official journal.

## Methods

### Study design and setting

This was a pre-post educational survey study conducted during the Journal of Abdominal Wall Surgery (JAWS) Workshop on Artificial Intelligence in Abdominal Wall Surgery at the 48th Annual Congress of the European Hernia Society (EHS), held in Porto, Portugal, from June 3 to 5, 2026. The workshop was designed as a focused 90-min educational session on the responsible use of generative AI in scientific publishing. Its objectives were to introduce current applicatinos of AI in scientific writing, discuss its main technical and ethical limitations, and address its responsible use in manuscript preparation and peer-review. Clinical applications of AI in abdominal wall surgery were not a primary focus and were addressed only indirectly when relevant to the discussion.

The workshop audience consisted of surgeons and surgical trainees attending a specialty congress focused on hernia and abdominal wall surgery. Many attendees were clinicians with a substantial professional interest or practice in abdominal wall surgery, including members of the EHS community and individuals involved in abdominal wall research and scientific publishing. However, individual data on surgical subspecialisation, FEBS-AWS certification, career stage, or editorial roles were not systematically collected.

A baseline survey was completed before the educational intervention, and a final survey was completed after the intervention. The educational intervention consisted of two presentations focused on (1) current knowledge and practical use of AI by early-career surgeons in scientific writing and (2) the perspective of a scientific publisher/editorial process regarding AI use in manuscript preparation and peer-review. The sessions addressed potential benefits, technical limitations, hallucinations, fabricated references, bias, confidentiality, authorship, disclosure, peer-review implications and the need for professional guidance.

This was a convenience sample of workshop attendees who voluntarily completed both surveys. No *a priori* sample size calculation was performed because the study was designed as a pragmatic educational evaluation of a single workshop, and all attendees were invited to participate. Each participant was asked to enter an anonymous code or nickname in the first survey and to use the same code in the final survey to allow exploratory paired analysis while preserving anonymity. Each survey included 10 questions. Both questionnaires were developed with the assistance of ChatGPT Plus, and were informed by previously validated instruments (the Kirkpatrick Questionnaire and the Technology Acceptance Model) [13, 14] and adapted to the use of AI in the context of our workshop.

This was a pragmatic educational intervention. The baseline survey assessed previous knowledge and use of AI in scientific writing, including tools such as ChatGPT or Copilot, purposes of use, perceived productivity, awareness of fabricated references, trust without verification, disclosure, AI as a potential co-author, confidentiality concerns and need for additional training. The post-workshop survey was not designed as a direct replication of every baseline item. Instead, it assessed post-intervention knowledge, understanding of AI limitations, ability to identify risks in abdominal wall surgery, intended changes in AI use, reference verification, disclosure, ethical/legal understanding, perceived need for society guidelines, preparedness for responsible use, and overall workshop impact. The item addressing specialty-specific AI risks was included only in the post-workshop survey as an exploratory assessment of participants’ perceived ability to translate the general principles discussed during the session to the abdominal wall surgery context.

The post-workshop questionnaire was intentionally designed to evaluate educational outcomes rather than simply repeat the baseline assessment. Consequently, while both surveys covered the same conceptual domains, only the knowledge item used identical response categories and could be directly compared. The remaining domains were assessed using complementary questions intended to capture changes in understanding, attitudes, and intended behaviour following the educational intervention.

### Outcomes

The primary outcome was the change in self-reported knowledge of AI in scientific publishing and writing. Secondary outcomes included baseline AI use, perceived risks, attitudes toward transparency, confidentiality concerns, post-workshop understanding of AI limitations, intended reference-verification practices, support for guidelines, preparedness for responsible use, and perceived workshop impact.

### Statistical analysis

Categorical variables are reported as frequencies and percentages. Multiple-response items were analysed by counting each selected option independently; therefore, percentages for those items can sum to more than 100%. Anonymous participant codes were normalized by removing spaces and converting them to lowercase. Matched analysis was performed only for the knowledge item because the response options were directly comparable between surveys. Knowledge categories were scored ordinally as none = 0, basic = 1, intermediate = 2, and advanced = 3. The Wilcoxon signed-rank test was used for the matched pre-post comparison. Because most other items differed in wording and response structure, they were analysed descriptively by thematic domain rather than by inferential pre-post testing. Analyses were performed using Python 3.11.

ChatGPT-5.5 was used to assist with language drafting, document formatting and the graphical development of the alluvial plot from author-verified study data. Generative AI was not used for statistical analysis or to generate or modify study data or results.

## Results

### Survey completion and participant matching

A total of 39 participants completed the baseline survey and 41 completed the final survey. After normalization of anonymous codes, 34 participants could be matched across surveys. Five baseline codes were not present in the final survey, and seven final-survey codes were not present in the baseline survey, including one respondent who explicitly indicated that they had not completed the first survey.

### Baseline knowledge and AI use

Baseline response distributions are shown in Table 1. Before the workshop, 4/39 participants (10.3%) reported no knowledge of AI in scientific writing, 19/39 (48.7%) basic knowledge, 14/39 (35.9%) intermediate knowledge, and 2/39 (5.1%) advanced knowledge. Most respondents had already used AI tools: 14/39 (35.9%) occasionally, 14/39 (35.9%) frequently, and 7/39 (17.9%) systematically, whereas 4/39 (10.3%) had never used them. The most common applications were language editing (29/39, 74.4%), manuscript structuring (18/39, 46.2%), literature summarization/review (18/39, 46.2%), and data analysis or interpretation (16/39, 41%).

**TABLE 1:** Baseline survey response distributions (n = 39). * Multiple selections were allowed.

Item	Response	n (%)
What is your level of knowledge regarding the use of AI in scientific writing?	None	4 (10.3)
	Basic	19 (48.7)
	Intermediate	14 (35.9)
	Advanced	2 (5.1)
Have you used AI tools (ChatGPT, Copilot, etc.) for scientific activities?	Never	4 (10.3)
	Occasionally	14 (35.9)
	Frequently	14 (35.9)
	Systematically	7 (17.9)
For what purposes have you used AI?*	Language editing	29 (74.4)
	Manuscript structuring	18 (46.2)
	Summarizing/literature review	18 (46.2)
	Data analysis or interpretation	16 (41)
	None of the above	4 (10.3)
AI improves scientific productivity	Strongly disagree	1 (2.6)
	Disagree	6 (15.4)
	Agree	23 (59)
	Strongly agree	9 (23.1)
AI may generate errors or fabricated references	Unlikely	0
	Possible	15 (38.5)
	Frequent	13 (33.3)
	Very frequent	11 (28.2)
Do you trust AI-generated scientific content without additional verification?	Yes	1 (2.6)
	Only partially	8 (20.5)
	Rarely	15 (38.5)
	Never	15 (38.5)
The use of AI should be declared in scientific manuscripts	No	0
	Only in certain cases	9 (23.1)
	Yes	7 (17.9)
	Yes, mandatorily and in a standardized manner	23 (59)
AI can be considered a co-author	Yes	2 (5.1)
	No	25 (64.1)
	Only in certain cases	2 (5.1)
	I do not know	10 (25.6)
Are you concerned about confidentiality when uploading manuscripts or peer-reviews to AI platforms?	Not at all	4 (10.3)
	Slightly	15 (38.5)
	Moderately	9 (23.1)
	Very much	11 (28.2)
Do you believe you need more training in AI applied to scientific publishing?	No	1 (2.6)
	Probably not	1 (2.6)
	Probably yes	18 (46.2)
	Definitely yes	19 (48.7)

### Baseline attitudes toward productivity, risks, disclosure, authorship, confidentiality, and training

At baseline, 32/39 respondents (82.1%) agreed or strongly agreed that AI improves scientific productivity. All respondents considered AI-generated errors or fabricated references at least possible, and 24/39 (61.5%) rated them as frequent or very frequent. Trust without additional verification was low: 30/39 (76.9%) reported rarely or never trusting AI-generated scientific content without verification. Disclosure was widely supported, with 30/39 (76.9%) selecting either yes or mandatory standardized disclosure. Most respondents rejected AI co-authorship (25/39, 64.1%), although 10/39 (25.6%) were uncertain. Participants perceived several benefits while also recognizing some of the potential risks. Confidentiality concerns were common, with 35/39 (89.7%) reporting at least slight concern and 20/39 (51.3%) reporting moderate or very high concern. Almost all participants (37/39, 94.9%) believed more training was probably or definitely needed.

### Post-workshop knowledge, risk awareness, and responsible-use intentions

Post-workshop response distributions are shown in Table 2. After the workshop, 26/41 respondents (63.4%) rated their knowledge as intermediate and 2/41 (4.9%) as advanced. Thirty-eight participants (92.7%) agreed or strongly agreed that they now had a better understanding of technical limitations such as hallucinations, bias, and fabricated references. Seventeen participants (41.5%) answered yes or clearly yes to being able to identify specific risks of AI use in abdominal wall surgery, while 21/41 (51.2%) answered partially.

**TABLE 2:** Final survey response distributions after the workshop (n = 41).

Item	Response	n (%)
After this workshop, how would you rate your knowledge of AI in scientific publishing?	None	2 (4.9)
	Basic	11 (26.8)
	Intermediate	26 (63.4)
	Advanced	2 (4.9)
I now have a better understanding of the technical limitations of AI (hallucinations, bias, fabricated references)	Strongly disagree	1 (2.4)
	Disagree	2 (4.9)
	Agree	30 (73.2)
	Strongly agree	8 (19.5)
I am able to identify specific risks of AI use in abdominal wall surgery	No	3 (7.3)
	Partially	21 (51.2)
	Yes	13 (31.7)
	Clearly yes	4 (9.8)
I will modify the way I use AI after this session	Not at all	6 (14.6)
	Slightly	16 (39)
	Moderately	14 (34.1)
	Significantly	5 (12.2)
I will systematically verify references generated by AI.	No	0
	Sometimes	1 (2.4)
	Almost always	7 (17.1)
	Always	33 (80.5)
I believe that the use of AI must be declared in scientific manuscripts	No	0 (0.0)
	In some cases	6 (14.6)
	Yes	6 (14.6)
	Yes, with clear regulation	29 (70.7)
I now better understand the ethical and legal implications of AI use	No	1 (2.4)
	Partially	7 (17.1)
	Yes	22 (53.7)
	Clearly yes	11 (26.8)
I believe scientific societies should develop specific guidelines on AI use	No	1 (2.4)
	Optional	4 (9.8)
	Recommended	15 (36.6)
	Essential	21 (51.2)
I feel more prepared to use AI responsibly	Not at all	0
	Slightly	9 (22.0)
	Moderately	23 (56.1)
	Significantly	9 (22)
Overall evaluation of the workshop	Low impact	1 (2.4)
	Moderate impact	13 (31.7)
	High impact	21 (51.2)
	Very high impact	6 (14.6)

Most participants reported intended practice changes after the session: 16/41 (39%) would modify their AI use slightly, 14/41 (34.1%) moderately, and 5/41 (12.2%) significantly. Reference verification was the strongest post-workshop responsible-use signal: 33/41 respondents (80.5%) stated they would always verify references generated by AI. Disclosure remained strongly supported with 35/41 (85.4%) selecting yes or yes with clear regulation. Most participants (80.5%) reported a better understanding of the ethical and legal implications of AI use, answering “yes” or “clearly yes”. Similarly, 87.8% considered specific guidance from scientific societies to be recommended or essential. Overall, 32 of 41 respondents (78.0%) reported feeling moderately or highly prepared for the responsible use of AI.

### Matched knowledge analysis

Among the 34 matched participants, the median ordinal knowledge score increased from basic (median score 1, IQR 1-2) before the workshop to intermediate (median score 2, IQR 2-2) after the workshop. Knowledge improved in 18/34 participants (52.9%), remained unchanged in 13/34 (38.2%), and was lower in 3/34 (8.8%). Overall, the paired comparison showed a significant improvement in self-reported knowledge (Wilcoxon signed-rank test, p = 0.0005). Detailed matched transitions are presented in Figure 1.

**FIGURE 1:**
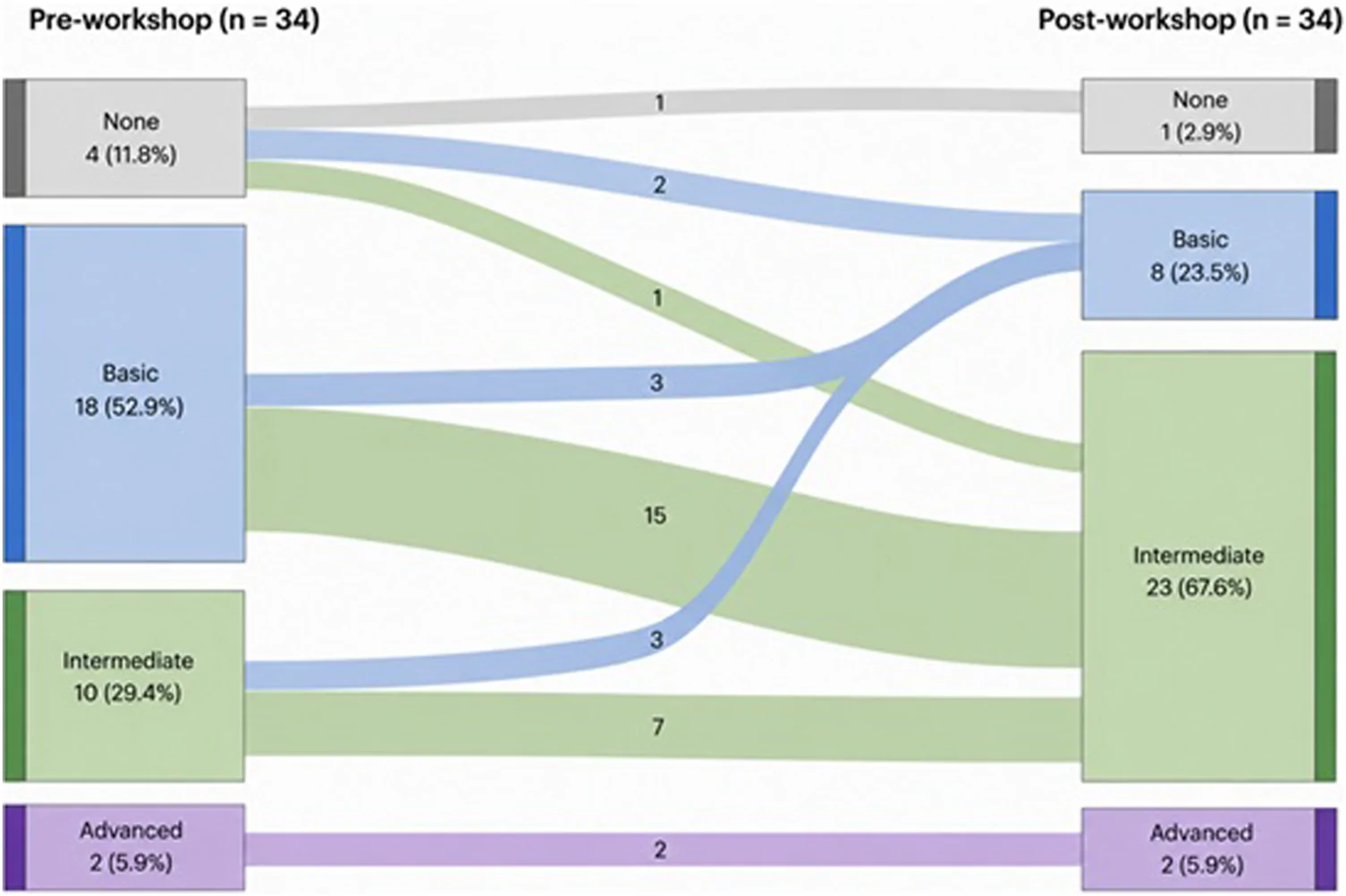
Alluvial plot illustrating individual transitions.

### Overall workshop evaluation

Overall perceived educational impact was assessed separately from change in self-reported knowledge. Twenty-seven participants (65.8%) rated the overall impact of the workshop as high or very high, 13 (31.7%) as moderate, and one (2.4%) as low.

## Discussion

The present study suggests that generative AI is no longer an emerging technology in scientific publishing, with its use already common among participants attending this workshop. This pre-post educational survey shows that a focused workshop on AI in scientific publishing was associated with an improvement in self-reported AI knowledge among participants. The most direct evidence was the matched knowledge analysis, in which more than half of paired respondents moved to a higher ordinal self-reported knowledge category. Although the intervention was intentionally brief, the improvement was accompanied by strong post-workshop signals of responsible-use intentions, particularly systematic verification of AI-generated references and support for disclosure and guideline development.

At baseline, nearly 90% of respondents had already used AI tools for scientific activities, most commonly for language editing. These findings suggest that generative AI is already being incorporated into the academic activities of many participants in this selected group. This pattern is consistent with published discussions of generative AI-assisted academic writing, which emphasize potential benefits in drafting, editing, translation, readability and organization while warning that the final scientific responsibility remains with human authors [4, 15]. For many participants, AI was used as a tool for improving the clarity and efficiency of scientific writing. Nevertheless, our findings suggest that this widespread adoption has not always been matched by a clear understanding of its limitations and risks particularly with respect to verification, appropriate use and confidentiality.

All respondents considered errors or fabricated references at least possible and most reported low trust in AI-generated scientific content without verification. Empirical work has shown that LLMs can generate references with low precision and high hallucination rates when prompted for systematic review tasks, supporting the need for verification [6]. Recent studies on AI hallucinations have shown that the apparent fluency and credibility of AI-generated text do not guarantee its accuracy. Such outputs may contain factual errors, fabricated references or conclusions that are not supported by the underlying evidence [16]. Recent editorial experience suggests that this risk is already reaching the scientific literature: the JAMA Network has reported receiving manuscripts containing inaccurate or fabricated references and has consequently updated its guidance to discourage the use of generative AI for generating or managing bibliographic Ref. [7]. Our finding that 97.6% of participants reported that they would almost always or always verify AI-generated references suggests that even a brief educational intervention can reinforce good scientific practice by encouraging systematic verification.

Transparency emerged as another central domain [7]. At baseline, most participants supported declaration of AI use in manuscripts and after the workshop, 85.4% supported disclosure of AI use and regulation. This aligns with WAME and COPE (Committee on Publication Ethics) recommendations that AI tools cannot be listed as authors because they cannot assume accountability, but their use should be disclosed when they contribute to manuscript preparation [8, 17]. The distinction between assistance and authorship is especially relevant for early-career surgeons who may perceive AI tools as collaborators rather than instruments. Educational programs should therefore emphasize that authorship remains a human responsibility involving accountability for design, data integrity, interpretation, conclusions and responses to peer-review.

The workshop also addressed peer-review and confidentiality. Baseline confidentiality concern was substantial with more than half of participants reporting moderate or very high concern when uploading manuscripts or peer-reviews to AI platforms. Generative AI has begun to affect peer-review, but journal policies frequently restrict uploading confidential manuscripts or reviewer reports into third-party AI systems unless explicitly allowed and disclosed [9]. Reviews of AI use in peer-review describe potential benefits in efficiency, screening, and quality control, while also emphasizing concerns related to confidentiality, bias, transparency, and erosion of expert judgment [10]. The baseline concern regarding confidentiality when uploading manuscripts or peer-reviews to AI platforms highlights the relevance of addressing peer-review-specific issues in future AI education. This is particularly relevant because peer-review materials are confidential and should not be uploaded to external AI systems unless explicitly permitted by journal policy [7].

The need for responsible AI literacy is particularly relevant to abdominal wall surgery, where AI and machine-learning methods are increasingly being investigated for complication and recurrence prediction, operative workflow recognition, and abdominal wall reconstruction planning [2]. As surgeons become both users and producers of AI-related research, they need competence not only in clinical AI concepts but also in how AI can and cannot be used to write, review and publish.

Despite the positive educational impact of the workshop less than half (41.5%) of participants felt confident in identifying AI-related risks specific to abdominal wall surgery. A small number of participants reported low post-workshop ratings, including two respondents who continued to rate their knowledge as “none” and one who strongly disagreed that their understanding of AI limitations had improved. Interestingly, three participants rated their AI knowledge lower after the workshop. The reasons for these responses cannot be determined from the survey. They may reflect differences in workshop attendance or engagement, interpretation of the questions, or a more critical reassessment of prior knowledge following the session. These findings are not surprising as the workshop was primarily designed to address the responsible use of AI in scientific publishing rather than its clinical applications. Nevertheless, these findings highlight the need for future educational initiatives to complement publication-focused AI literacy with specialty-specific training. Case-based discussions covering hernia surgery, abdominal wall reconstruction, clinical prediction models, image analysis and AI-assisted operative video assessment would seem a natural next step.

The high perceived need for training at baseline and strong post-workshop support for society guidelines have practical implications. Surgical societies are well positioned to produce specialty-specific guidance that translates general publication ethics into operational recommendations: acceptable AI uses, required disclosure language, reference-verification workflows, confidentiality safeguards, authorship boundaries, peer-review restrictions and minimum competencies for trainees. Broader reviews in medical and surgical education similarly identify the need for structured AI curricula, faculty development and validated educational outcomes rather than *ad hoc* adoption [18, 19].

This study has several limitations. First, this was a single-workshop survey with a modest, voluntary sample and no control group, limiting the generalizability of the findings. Although the workshop was conducted within a specialty abdominal wall surgery congress, individual professional characteristics and the degree of abdominal wall specialization were not systematically recorded. Therefore, the sample cannot be assumed to represent all abdominal wall surgeons. Second, outcomes were self-reported and measured immediately after the intervention; therefore, the study does not demonstrate long-term retention or actual behavioural change. Third, although anonymous codes allowed matching for 34 participants, not all responses could be paired. Fourth, the baseline and final surveys intentionally differed in several domains, limiting formal item-by-item pre-post comparisons. Fifth, no demographic variables were collected; therefore, subgroup analyses by age, career stage, language background, editorial experience, or previous AI exposure were not possible. In addition, not all survey items included a neutral or “I do not know” response option, which may have encouraged respondents to select a directional response and may have influenced the distribution of reported attitudes. Finally, the study assessed perceptions rather than objective AI-literacy performance. Future studies should use validated AI literacy instruments, objective knowledge tests, follow-up surveys and behavioural outcomes such as accuracy of reference checking or quality of AI-use disclosure statements. Despite these limitations, this study provides a practical model for integrating responsible AI training into surgical education.

### Summary comments

Among voluntary participants attending an AI-focused workshop at an international abdominal wall surgery congress, previous use of AI for scientific activities was common, and the educational intervention was associated with an increase in self-reported knowledge among matched respondents, while participants also expressed support for key principles of responsible AI use. Although these findings cannot be generalized to the broader abdominal wall surgery community, educational initiatives such as this workshop provide a practical model for supporting the responsible integration of AI into scientific publishing and may represent a first step towards developing AI-literate authors, reviewers, and editors in this field. As AI continues to evolve, scientific societies and journals have an important opportunity to lead this transition by promoting education, transparency, and integrity alongside technological innovation.

## Data Availability

The raw data supporting the conclusions of this article are available from the corresponding author upon reasonable request.
